# Development of a decision aid for cardiopulmonary resuscitation and invasive mechanical ventilation in the intensive care unit employing user-centered design and a wiki platform for rapid prototyping

**DOI:** 10.1371/journal.pone.0191844

**Published:** 2018-02-15

**Authors:** Ariane Plaisance, Holly O. Witteman, Annie LeBlanc, Jennifer Kryworuchko, Daren Keith Heyland, Mark H. Ebell, Louisa Blair, Diane Tapp, Audrey Dupuis, Carole-Anne Lavoie-Bérard, Carrie Anna McGinn, France Légaré, Patrick Michel Archambault

**Affiliations:** 1 Department of Social and Preventive Medicine, Faculty of Medicine, Université Laval, Québec, QC, Canada; 2 Centre intégré en santé et services sociaux de Chaudière-Appalaches, Lévis, QC, Canada; 3 Centre de recherche sur les soins et les services de première ligne de l’Université Laval, Institut universitaire de première ligne en santé et services sociaux, Université Laval, Québec, QC, Canada; 4 Office of Education and Continuing Professional Development, Faculty of Medicine, Université Laval, Québec, QC, Canada; 5 Population Health and Optimal Health Practices Research Group, CHU de Québec-Université Laval Research Centre, Québec, QC, Canada; 6 Department of Family Medicine and Emergency Medicine, Faculty of Medicine, Université Laval, Québec, QC, Canada; 7 School of Nursing and Centre for Health Services and Policy Research University of British Colombia, Vancouver, BC, Canada; 8 Clinical Evaluation Research Unit, Kingston General Hospital, Kingston, ON, Canada; 9 Department of Critical Care, Queen’s University, Kingston, ON, Canada; 10 Department of Epidemiology and Biostatistics, College of Public Health, University of Georgia, Athens, GA, United States of America; 11 Faculty of Nursing, Université Laval, Québec, QC, Canada; 12 Institut universitaire de cardiologie et pneumologie de Québec, Québec, QC, Canada; 13 Department of Information and Communications, Faculty of Arts and Human Sciences, Université Laval, Québec, QC, Canada; 14 Faculty of Medicine, Université Laval, Québec, QC, Canada; 15 Department of Anesthesiology and Critical Care Medicine, Intensive Care Division, Faculty of Medicine, Université Laval, Québec, QC, Canada; UNITED STATES

## Abstract

**Background:**

Upon admission to an intensive care unit (ICU), all patients should discuss their goals of care and express their wishes concerning life-sustaining interventions (e.g., cardiopulmonary resuscitation (CPR)). Without such discussions, interventions that prolong life at the cost of decreasing its quality may be used without appropriate guidance from patients.

**Objectives:**

To adapt an existing decision aid about CPR to create a wiki-based decision aid individually adapted to each patient’s risk factors; and to document the use of a wiki platform for this purpose.

**Methods:**

We conducted three weeks of ethnographic observation in our ICU to observe intensivists and patients discussing goals of care and to identify their needs regarding decision making. We interviewed intensivists individually. Then we conducted three rounds of rapid prototyping involving 15 patients and 11 health professionals. We recorded and analyzed all discussions, interviews and comments, and collected sociodemographic data. Using a wiki, a website that allows multiple users to contribute or edit content, we adapted the decision aid accordingly and added the Good Outcome Following Attempted Resuscitation (GO-FAR) prediction rule calculator.

**Results:**

We added discussion of invasive mechanical ventilation. The final decision aid comprises values clarification, risks and benefits of CPR and invasive mechanical ventilation, statistics about CPR, and a synthesis section. We added the GO-FAR prediction calculator as an online adjunct to the decision aid. Although three rounds of rapid prototyping simplified the information in the decision aid, 60% (n = 3/5) of the patients involved in the last cycle still did not understand its purpose.

**Conclusions:**

Wikis and user-centered design can be used to adapt decision aids to users’ needs and local contexts. Our wiki platform allows other centers to adapt our tools, reducing duplication and accelerating scale-up. Physicians need training in shared decision making skills about goals of care and in using the decision aid. A video version of the decision aid could clarify its purpose.

## Introduction

Death in intensive care units (ICU) is common and most are preceded by a decision to withhold or to withdraw life-sustaining therapies [1–3]. Three decades of research have highlighted major communication failures on this issue between clinicians and patients or their family members [4–6]. Communication breakdowns can lead to patients dying in a distressed state while receiving aggressive life-sustaining interventions [7]. Upon admission to an intensive care unit (ICU), all patients should discuss their goals of care [5, 7]. Without such discussions, interventions that prolong life at the cost of decreasing its quality may be used without informed guidance from patients [5–9].

Clinical practice guidelines recommend shared decision making (SDM) to facilitate discussions about goals of care and the desirability of aggressive life-sustaining interventions [9]. SDM involves health professionals and patients making decisions together based on the best available evidence, health professionals’ experience, and patients’ values and preferences [10–12]. Decision aids (DAs) can help clinicians engage in SDM with their patients [12]. Routinely engaging patients and their family members in discussions about both options (i.e. forgoing or pursuing life-sustaining interventions) recognizes patient autonomy, improves the experience of dying, and reduces family distress [13]. Although DAs about life-sustaining interventions exist and can be found online on websites such as the A to Z Inventory of Decision Aids [14], these DAs are not universally used in hospitals around the world. Lack of adaptation to local contexts, cultures and patient needs is one barrier that impedes use of these tools.

Wikis are websites that allow users to contribute or edit content directly from a web browser [15]. Wikis have been used with some success in the creation of knowledge tools adapted to the needs of patients and healthcare professionals [16, 17]. However, to the best of our knowledge, wikis have never been used to engage knowledge users in adapting existing DAs to local contexts and to the specific needs of users (patients and clinicians) [18]. For these reasons, our primary objective was to engage end-users in adapting an existing decision aid about CPR to create a locally-adapted wiki-based decision aid about goals of care that could be individually tailored to a patient’s risk factors. The secondary objective was to document the use of a wiki platform for this purpose.

## Methods

### Clinical context and population

This study took place in a closed medical and surgical ICU with 18 beds and six critical care specialists in Levis (Canada). The last author (PA) works as an intensivist in this ICU. This unit treats medical, surgical, trauma, and obstetric patients. The unit also admits intermediate care patients. In 2015, 1092 patients were admitted (mean age 63.6 years, mean length of stay 3.8 days). On average, 20% of patients admitted are mechanically ventilated at some point during their stay. The annual mortality rate in the ICU is 7%.

For our study, eligible patients were alert and capable adults (≥ 18 years) admitted to the ICU. They were excluded if considered unstable in the intensivists’ judgement, were intubated, had cognitive impairment, or did not speak French.

We employed a two-phase user-centered design [19] to involve end-users (patients, family members and clinicians such as intensivists and allied health professionals) to adapt our DA to the local context (S1 Fig). Our study was approved by the Ethics Committee of the *Centre intégré de santé et de services sociaux de Chaudière-Appalaches*. A detailed description of our research protocol can be found elsewhere [20] and S1 Text.

### Phase I: Needs assessment

#### 1.Ethnographic observations in ICU

Between May and June 2015, the principal author, trained in qualitative research methods as an anthropologist (AP), conducted three non-consecutive weeks of ethnographic observations of daily interactions among patients, families, intensivists and other allied health professionals in the ICU to enrich understanding of the context [21].

#### 2.Observation of discussions about goals of care

After obtaining verbal consent from patients and their intensivists, the principal author observed five dyads of attending intensivists and their adult ICU patients, with family members if present, discussing goals of care and the potential use of CPR and/or invasive mechanical ventilation. She noted users’ needs, goals, strengths, limitations, intuitive decision-making processes and the discussion surroundings using an observation grid created by a human factors engineer (HOW) translated and adapted to our context (S2 Text and S3 Text)

#### 3. Semi-structured interviews

She then conducted semi-structured interviews with five of the six intensivists. The interview grid was informed by the ethnographic data and questions were about the role intensivists perceived they play in decision-making about goals of care and whether patients’ decisions about goals of care are adequately informed (S4 Text).

#### 4. Content analysis

Two researchers (AP, PMA) performed content analysis of the field notes and transcribed the interviews verbatim using the R package for Qualitative Data Analysis, version 0.2–7.

### Phase II: Prototype development

#### 1.Creation of wiki

Using Dokuwiki and its GNU General Public Licence, we created a wiki that presented our project and archived the different versions of our DA for future use and adaptation to other contexts.

#### 2. Development of prototype

We found the paper-based “Cardiopulmonary Resuscitation (CPR) Decision Aid for Patients and Their Families” [22] using the keyword “CPR” in the A to Z Inventory of Decision Aids [14]. This was the only free Canadian DA relevant to our context and for which we were granted translation and adaptation permission. This English-only DA presents population-level statistics about CPR outcomes classified by age and by reason for cardiac arrest. It met 15 of the 22 applicable criteria of the International Patient Decision Aid Standards (IPDAS) Collaboration [23]. Authors used various quantitative scales to measure patients’ and family members’ opinions about the usefulness, social acceptability, and neutrality of the information presented in the DA [24]. We translated this DA into French [25].

#### 3. Development of the first GO-FAR score calculator prototype

The *Good Outcome Following Attempted Resuscitation* (GO-FAR) clinical prediction rule estimates the likelihood of neurologically intact survival after in-hospital cardiopulmonary resuscitation (S1 Table and S2 Table). We hypothesized that this new clinical prediction rule would be a useful component of a shared decision-making process regarding goals of care [26]. The author (MHE) granted us permission to use the programming code. We translated the GO-FAR score into French and embedded the calculator in our wiki.

#### 4. Rapid prototyping

We enrolled five patients and family members (if available) in each of three rapid prototyping cycles. We estimated that a sample of at least 15 participants would be adequate to detect over 90% of all usability problems [27]. Once the principal author obtained written consent, participants were given a minimum of three hours to read the DA prototype and then discussed it with the principal author and the attending intensivist. The latter also presented the online GO-FAR calculator prototype to the patient either on an iPad or an iPhone (Apple Inc, Cupertino, CA, USA). The principal author recorded the discussion and noted users’ needs, goals, strengths, limitations, and intuitive processes of decision making with the use of the DA, using the same observation grid as in phase I. Then she conducted interviews with participants regarding their experience of using the DA (i.e., clarity, social acceptability, relevance of the information presented, preferred elements and improvements to be made). She also collected intensivists’ feedback about each DA prototype.

### Data collection for Phase II

We collected sociodemographic characteristics of participants and intensivists. Even though our project did not aim to change patients’ goals of care, we hypothesized that they might change their goals of care or form them for the first time as a result of participating in the research project. We therefore retrospectively documented the existence and the content of official level-of-care forms (i.e., form documenting the patient’s goals of care and advance directives concerning CPR and invasive mechanical ventilation) in the patient’s chart at ICU admission and discharge.

### Data analysis for Phase II

After each rapid prototyping cycle, two researchers (AP and PMA) performed qualitative content analysis of the transcripts, audio recordings, interviewer notes, and observations to identify usability problems and need for content clarification. They then made changes to the prototype and presented it to the next participants.

## Results

### Phase I: Needs assessment

The three weeks of ethnographic observations of daily interactions among patients, families, intensivists and other allied health professionals in the ICU led to many changed in the original DA (Table 1).

**Table 1:** Needs assessment results and changes made to the original DA.

Needs	How the needs were expressed	Changes
Need for information about invasive mechanical ventilation	During goals of care discussions, invasive mechanical ventilation was frequently discussed and patients struggled with decisions about this intervention.	Section added about invasive mechanical ventilation (see the DA in S5 Text) using information from another DA (used with permission) about prolonged mechanical ventilation.
Need for information about patient’s functional autonomy prior to admission to the ICU and level of functional decline that patients would deem acceptable at discharge	*Intensivist*: “I like to ask my patient: ‘What was your functional autonomy before this acute illness? Would you accept not being able to go back home after your stay in the ICU?’ It helps to open up the conversation and know which interventions should be offered.”	Questions added about patient’s functional autonomy prior to ICU admission and level of functional decline they would deem acceptable at discharge (S5 Text).
Need for simple and clear information	When speaking to patients, intensivists tend to: - Avoid using taboo words such as “death”: e.g., *“*Are you so tired of the [non-invasive] mask that you would like us to take it off and let you go?” The expression “let you go” is a euphemism for “let you die” (*“vous laisser mourir”*). - Use medical jargon: e.g., “Your chart states you want to be Level 1, is this right?” (instead of saying: do you want full resuscitation?). - Minimize complex interventions (such as CPR) and their consequences: “If your heart stops beating, do you want us to massage it?”	Words used that were clear and did not leave any room for misunderstanding by patients about death, dying, and the invasive nature of CPR.

Originally, we had planned to build the DA directly on the wiki platform and ask patients to participate in its creation using the wiki. However, it became clear during Phase 1 that our targeted population was unable to manage an iPad or any other electronic device. As a result, for Phase II, we created a paper version of the DA and asked participants to provide immediate verbal feedback on the prototype. The wiki was used instead as a knowledge management and dissemination platform and for programming the online GO-FAR prediction rule, which became an online complement to the DA that intensivists could use at their discretion.

### Phase II: Development phase

We modified the translated DA according to needs observed in Phase I and to as many IPDAS criteria [23] as possible. As planned in our protocol, we integrated the GO-FAR prediction rule into the wiki. We presented the first prototype to the six participating intensivists, as we envisaged they would present the DA to patients prior to a face-to-face discussion. They all agreed with the content but insisted that the ICU nurses should also approve the DA before it was shown to patients. The three nurse leaders invited to participate (head ICU nurse, assistant-head ICU nurse, ICU nurse educator) feared that a DA would *replace* a physician-led discussion about goals of care. They were also apprehensive that anxious patients would be asking the nurses more questions raised by the DA. They also feared that the DA would negatively influence healthy and fit patients into refusing CPR and invasive mechanical ventilation even when it was appropriate. Finally, they felt that words such as “death” and “induced coma” were too grim to be used in the DA. In response to these concerns, we sought a delicate balance between using precise words to describe CPR and invasive mechanical ventilation and their risks (including death, pain and suffering) and using more socially acceptable words. After changing the DA (Table 2) and reassuring nurses it would never replace in-person conversations, we received their full support for presenting the prototype to the first five participants.

**Table 2:** Modifications to the prototype following comments by nurse leaders.

Nursing leaders’ rationale	Modifications
Nurses were not comfortable about transmitting uncertainty to patients about the potential of dying after attempted CPR.	We removed the words “to try” in the following sentence: “Cardiopulmonary Resuscitation (CPR) is the term used to describe the treatments used **to try** to restart a person’s heart after it has stopped beating.”
The French-Canadian idiomatic expression “*être branché sur une machine*” [“to be hooked up to a machine”] used to describe invasive mechanical ventilation was perceived as potentially confusing for patients because it could also mean being connected to a non-invasive ventilator or to a dialysis machine.	We replaced the expression *“être branché sur une machine”* [“to be hooked up to a machine”] with the exact medical term “ventilation mécanique invasive” [invasive mechanical ventilation”] and added the popular term in brackets “être branché sur une machine respiratoire” [being connected to a breathing machine].
There was general discomfort with the statistics presented about CPR survival in our ICU, such as a survival to discharge rate of 18% for the general population and of 2% for critically ill patients, with half of the survivors being discharged to a nursing home. Nurses believed that this information was too grim to be written in a DA. They also thought it could be dangerous because it could negatively influence healthy and fit patients into refusing CPR. Above all, they feared that patients would misinterpret these statistics without the expert support of a physician by their side to explain them.	We removed the section “How well does CPR work?” We replaced this section with an overall picture of CPR survival rates ranging from 0% to 30%. We also addressed nurses’ fears by presenting evidence from published studies. However, this also reinforced our decision to create a separate online GO-FAR calculator that intensivists could use at their discretion while discussing goals of care with patients to present more precise and specific survival predictions tailored to each patient.
Nurses perceived that our prototype was negatively biased toward influencing patients to refuse aggressive care. For example, in the following sentence in our first prototype, one of the advantages of the choice to receive CPR was *“*there is a small chance of returning home from hospital.” The word “small” was perceived as biased. We acknowledged the problem of using verbal descriptors to describe probabilities, given that “small chance” may be misinterpreted.	We changed the sentence for “there's a chance you won't return home from the hospital.”
Nurses felt that we were presenting invasive mechanical ventilation negatively. They felt that the wording “induced coma” should be replaced by “deep sleep” to describe the sedation required during invasive mechanical ventilation.	We changed “induced coma” for “deep sleep” in the section that describes the procedures necessary for invasive mechanical ventilation.

### Rapid prototyping

We invited 15 patients and six intensivists to participate in three rapid prototyping cycles (Table 3). In total, five patients/family members refused to participate in the rapid prototyping. One person stated that he/she did not want to participate in a research project while the four others were not comfortable with the subject of the research.

**Table 3:** Description of the participating patients and intensivists.

Patients (n = 15)	
Women, n (%)	8 (53)
Age, median (IQR)	69 (63–77)
Religion, n (%)	
Christian	12 (80)
None	2 (14)
Deist	1 (7)
High school not completed, n (%)	4 (27)
Reason for ICU admission	
Medical n (%)	13 (87)
Acute respiratory failure	2
Pneumonia	2
Leukemia treatment complication	2
Septic shock	2
Dieulafoy’s lesion	1
Disseminated zoster simplex infection	1
Gastrostomy complication	1
Suspected bowel obstruction	1
Overdose (accidental)	1
Surgical n (%)	2 (13)
Lung cancer	1
Pleuropericardial cyst	1
Length of stay in the ICU (days), median (IQR)	4 (3–6)
Intensivists (n = 6)	
Women, n (%)	2 (33)
Age, median (IQR)	38 (33–42)
Experience (number of years post-residency), median (IQR)	6.5 (2–11)
Fellowship in Critical Care, n (%)	6 (100)
Royal College of Physicians and Surgeons of Canada baseline speciality, n (%)	
Emergency medicine	2 (33)
Internal medicine	2 (33)
Anesthesiology	1 (17)
Respirology	1 (17)

### Prototyping cycles

We held the first cycle of rapid prototyping between July 15 and July 20, the second between July 22 and July 27, and the third between December 10 and 17, 2015. Table 4 presents the main needs identified, how they were expressed and the changes we made to the prototypes to address these needs. We reached saturation on the needs identified during each round of prototyping. The fourth column presents unmet needs that will need further solutions.

**Table 4:** Needs identified, needs met through changes to DA made during rapid prototyping, and unmet needs.

Needs	How they were expressed	Changes made	Further solutions
**First Cycle**			
Need for better statistics about the risk of losing functional autonomy following CPR and invasive mechanical ventilation.	Participant: *“*I want to know the details about my own risk of losing functional autonomy after a cardiac arrest.”	In the original GO-FAR paper, we could only calculate the probability of surviving attempted resuscitation for in-hospital cardiac arrest with a good neurological outcome (Cerebral Performance Category 1). Additional outcome data supplied by its author (MHE) enabled us to calculate the probabilities for each of the five Cerebral Performance Categories.	We were unable to find a prognostic prediction rule for the outcome of invasive mechanical ventilation. This should be developed in the future.
Need for better visual presentation of the outcome risks after attempted CPR	Intensivist: “So out of 100 patients like you, 18 will survive. But of these 18 survivors, only 9 will be able to return to live at home without major after-effects” Participant: “A 50% success rate? That’s still good!” Intensivist: “Well… it depends on how you see death, because if you include the patients who died, you only have a 9% success rate. . .”	We programmed the visual output for our online GO-FAR calculator to use the IconArray.com software using visual icons that are easier to understand for presenting the risks of being categorized in each of the five Cerebral Performance Categories after undergoing CPR (S2 Text).	
Need to illustrate the various possible consequences of over aggressive care.	Participant: “I thought that futile care meant continuing aggressive care when you are in a ‘vegetative state’. Now, I realize that it can also be about continuing aggressive care when a patient is completely conscious but has no more control over their body.”	We added a question about how patients would feel if they were “bedridden”.	
Need to clarify the hypothetical nature of the interventions in our DA (e.g., CPR in case of a sudden cardiac arrest)	Participant: “I don’t understand why you are asking me about this. My heart has always been all right and now you are telling me that my heart is going to stop?”	We clarified the introduction to make it clear that a patient’s heart can stop beating even if they don’t have a heart problem and that the decisions to be made were advance directives in case a cardiac arrest ever occurred.	
Need for a multimedia DA about CPR and invasive mechanical ventilation.	In discussion with a functionally quadriplegic patient with advanced muscular dystrophy, we realized that the patient could not hold our paper document in his hands and that we had to read the DA to him.		A video-based decision aid presented on a TV screen could be helpful for these patients.
**Second Cycle**			
Need for clarification about the alternatives to invasive mechanical ventilation.	Patients who did not understand the term non-invasive ventilation.	We presented non-invasive mechanical ventilation as a less effective alternative when invasive mechanical ventilation is needed.	Notwithstanding this modification, we still consider that physicians must adapt their vocabulary and improve their competencies in explaining complex interventions to patients.
Need for health system solutions to better document patients’ fundamental preferences about goals of care.	Some patients clearly knew that they did not want to be resuscitated or mechanically ventilated even though their medical chart indicated that they were “full code” status.		• Patients scheduled to be admitted to our ICU after major elective surgery will be targeted to receive our decision aid in the future. • We need centralized electronic patient records where such information can be stored and made available to all health professionals across the continuum of care.
Need for simple and clear information.	Medical resident: “People just don’t understand that if their heart stops beating and nothing is done they will die. We often need to explain really basic facts to patients. You always need to simplify information.”	We programmed our online calculator to automatically present output using icons to illustrate the outcome of cardiac arrest if nothing is done (i.e., 100 icons = 100% of patients will die) and the outcomes predicted by the GO-FAR rule if CPR is attempted (Appendix S9)	
**Third cycle**			
Need for simple and clear information.	• Participant: “I think your document is great for people who read, who are educated, but not for old people who are not well informed.” • 60% (n = 3/5) of the patients involved in the last cycle of rapid prototyping still did not understand the purpose of the DA.		However much we simplified the information in the DA, we realized that text explanations could only go so far to explain complex interventions, and that a video to complement our written DA would be needed.
Need to determine if SDM is the best approach for all patients with limited understanding.	SDM was difficult to apply with some patients. For example, one patient could not understand the questions addressed in the DA even after multiple explanations by the attending physician with and without our DA. This patient thought we were asking him for consent to surgery.		Further studies must be conducted about ways to adapt SDM to patients who are alert and capable, but cannot understand the decisions to be made.
Need to determine the role that patients prefer to play for decisions about goals of care.	Some patients refused to discuss goals of care because they were simply too uncomfortable or too emotional to talk about it.		Health professionals need more training to develop their communication skills to better adapt to a range of decision making roles (such as “informed non-dissent”, i.e. patients who prefer not to actively make a decision but only to assent or not to what the physician thinks appropriate)and discuss these topics with empathy and understanding.
Need to know more about the dying process of patients who survive CPR, but who do not leave the hospital alive.	Intensivist: “For my own ideal situation, CPR would be a success if I died straight away or if I fully recovered. What about those who die before they leave the hospital? When do they die? How long does it take for patients to die after attempted CPR for in-hospital cardiac arrest?		We were not able to obtain more precise outcome data about the dying experience for the patients in the GO-FAR study who did not survive. There is a need to know where, when and how these patients die to better understand the dying experience of the majority of patients who die before discharge after attempted CPR for in-hospital cardiac arrest.

### Documenting the use of a wiki platform for adapting DAs

#### Integrating the GO-FAR calculator into the wiki

We hired a MSc graduate student in computer programming, aided by two other programmers, to embed the GO-FAR calculator in the wiki. We used Dokuwiki, whose open-source programming architecture allowed us to embed the GO-FAR calculator using Visual Basic.Net converted to PHP syntax. It also allowed us to link the calculator to images created using IconArray.com (Risk Science Center and Center for Bioethics and Social Sciences in Medicine, University of Michigan, Ann Arbor, USA) to present the likelihood of survival after in-hospital cardiac arrest.

#### Wiki use during prototyping phases

For collecting comments from health professionals, we tried Google Slideshow (Google Inc., Mountain View, CA, USA) and Dokuwiki, but none were satisfactory. The latest paper prototype and online prediction calculator were always accessible on our wiki during the rapid prototyping cycles, however, and work is underway to keep it freely available permanently.

#### Wiki usage and editing statistics

We created the wiki on June 17, 2015, and a Google Analytics account to measure its use on September 5, 2015. Since then, www.wikidecision.org has been accessed 2174 times by 1554 users. The average length of stay is 68 seconds with a bounce rate of 77%. The bounce rate is the percentage of visits in which a visitor to the site accesses only the entrance page without clicking on any of the links on the page. Return visitors generated 620 sessions (29% percent of all sessions). Direct searches landing on our wiki homepage represent 735 sessions (34%). Lévis and Quebec City were the city of origin for 409 sessions (19%).

#### Presence/absence and content of official level-of-care forms

At the time of the study, our institution documented goals of care in a form called “Levels of care”. At Level 1, patients are considered to desire all life-sustaining interventions (including CPR and invasive mechanical ventilation). Patients who have no completed form signed by a medical doctor in their chart are also considered Level 1 *de facto*. At Level 2, patients receive interventions that prolong life but not at any cost (e.g., severe loss of neurological function). At Level 3, patients only receive comfort care such as pain and symptom relief.

We emphasized that we only wanted participants’ comments about the DA prototype and did not aim to change their documented level of care. Some patients, however, after discussing levels of care with our research team and learning about the advantages and disadvantages of CPR and invasive mechanical ventilation, asked to change their official level of care (Table 5). Scrutiny of the verbatim transcripts of the patient/intensivist discussions confirmed that modifications made to patients’ documented level of care were due to our intervention.

**Table 5:** Patients’ level of care upon ICU admission and discharge, any change and cause of change made.

Patient no.	Level of care upon ICU admission	Level of care upon ICU discharge	Change/cause of change
1	No form completed	No form completed	No change
2	Level 2	Level 2	No change
3	No form completed	No form completed	No change
4	No form completed	Level 2	Modified through the research process. Patient already had a clear and coherent choice against CPR.
5	No form completed	Level 2	Modified through the research process. Patient already had a clear and coherent choice against CPR.
6	No form completed	Level 2	Modified through the research process. Patient already had a clear and coherent choice against CPR.
7	Level 2	Level 2	No change
8	No form completed	Level 2	Modified through the research process. Patient already had a clear and coherent choice against CPR.
9	Level 1	Level 1	No change
10	Level 2	Level 2	No change
11	No form completed	No form completed	No change
12	No form completed	No form completed	No change
13	No form completed	No form completed	No change
14	No form completed	No form completed	The patient (aged 82 years) did not want CPR or invasive mechanical ventilation but this choice remained undocumented.
15	Level 1	Level 2	Modified through the research process. At first, this patient did not know much about CPR.

### Final decision aid

The final version of our paper-based DA and its wiki-based GO-FAR calculator met 18 of the 22 applicable IPDAS criteria for the first intervention (CPR) and 17 of the 22 applicable criteria for the second intervention (invasive mechanical ventilation) (S3 Table). The final version of the paper DA (S9) and the online GO-FAR prediction rule (S10) are freely available through www.wikidecision.org.

## Discussion

We developed a paper DA about goals of care in relation to two life-sustaining therapies used in the ICU (CPR and invasive mechanical ventilation). Based on observations of discussions between patients and intensivists about two DAs [22,26]-with input from their authors, we used user-centered design methodology to create a new DA adapted to the local context and the needs of patients and clinicians in an ICU in Quebec, Canada. As an adjunct, we combined the GO-FAR outcome prediction calculator with visual risk representation software to enable physicians to quickly tailor visual representations to individual patients to explain their chances of survival with a good neurological outcome after attempted CPR for in-hospital cardiac arrest. Our study results raised several questions about how levels of care are discussed and documented in our care setting and about DA development and user-centered design.

First, most patients felt the need to discuss goals of care with their attending intensivists even though this was not our goal. Ten of the 15 participating patients did not have their preferred level of care documented upon ICU admission. Six asked to complete or modify their official level of care form after discussing our DA, and six others left without completing a level of care form. This finding, that patients lacked documented goals of care, concur with those of Nouvet et al. who explored barriers to goals-of-care communication in Canada [28]. Even though physicians and nurses recognized the importance of discussing goals of care as early as possible with seriously ill patients, they also reported an unspoken norm whereby physicians and nurses delay such discussions until death is imminent and no technology can “save” the patient. They also reported fear of shocking the patient and cultural taboos around death and dying as two of the numerous barriers involved [28]. More research is needed about interventions to help physicians, nurses and critically ill patients to communicate about goals of care while the patients are still able to.

Second, this study raised some challenges in involving patients, doctors and other allied health professionals in user-centered design to create culturally appropriate DAs about sensitive subjects. We had to find a compromise between using lay language that patients and nurses suggested and using more medically precise language that doctors and researchers suggested. For example, in the popular language of Quebec, the expression *being hooked up to a machine* can refer to non-invasive ventilators and dialysis machines as well as invasive mechanical ventilators. We finally settled on using both lay and medical language. A related and even more complex issue that arose with user-centered design concerned talking about death. We had to find a compromise between presenting information in a way that left no room for patient misunderstanding about death and the invasive nature of CPR, and terms that were more acceptable to the nurses in our ICU, who shared a strong cultural taboo against talking directly about death or else did not agree that it was necessary. These compromises represent a major challenge to the goal of our DA, and indeed all DAs, which is to find simple, non-euphemistic ways of conveying the most reliable knowledge and to avoid over-simplifying complex decisions. Other DA developers employing user-centered design have also faced this challenge [28–31] It raises questions about the extent to which users should influence the information the DA aims to disclose, and who should have the final say. In our context, there is an increasing corpus of literature and governmental directives supporting the clarification of goals of care while the patient is still capable of expressing his/her wishes, however complex, emotionally charged and culturally sensitive the subject is [5–9]. The nurses’ reluctance to discuss death with patients in our study demonstrates the strength of social taboo in the face of awareness of official directives and even of one’s own values.

Third, this study raised challenges in designing DAs to match different care settings and different populations. The original DA was created for a hospital setting in Kingston, Ontario, for an English-speaking population with a different cultural background. Although it was created in the same country and with the input of health professionals, patients and family members [24], a direct translation from French to English proved insufficient because of differences in local contexts (ICU vs. inpatient medical unit), local culture and patients’ needs. Adapting knowledge tools such as DAs to local contexts is a critical process in knowledge translation. Yet little is known about scaling up this process so that more healthcare centers, and not just research teams, develop tools that better meet the needs of their users. In addition, while other researchers [16, 17] have used wikis with some success to facilitate collaboration with end-users in the creation of knowledge tools, our results did not bear this out. This may be because subjects in the ICU are unstable and their condition is life-threatening, while subjects studied by Gupta et al. (stable asthma patients) [16] and Van de Belt et al. (infertile couples) [17] were younger and not critically ill. In addition, busy intensivists did not have the time or training to edit wiki content. However, the wiki did allow easy mobile access to the online survival prediction calculator that was used by participating intensivists at the patient’s bedside.

Finally, our study underlines the challenges of designing decision aids for patients with low literacy and low health literacy in particular. Even after multiple efforts to simplify our version of the Kingston DA, 60% (n = 3/5) of the patients involved in the last cycle of rapid prototyping still did not understand the purpose of the DA. Indeed, some patients’ thought that they were going to be resuscitated or mechanically ventilated following the discussion, and others thought that we were asking them whether they wanted us to administer medically assisted death. The Quebec Act respecting end-of-life care, which legalized and framed physician-assisted dying, came into force on December 10, 2015, just as we were completing Phase 3 of our study, and this may have caused confusion among our participants. However, our experience does suggest that explaining the difference between limiting the intensity of care and medically assisted dying is a challenging new responsibility for ICU physicians in countries where this has been legalized.

Our study has limitations. First, all of our user testing took place at a single site, all in French, and with a small number of participants recruited by the principal author. Our DA is now context- and culturally-adapted to a Caucasian French Canadian and mostly Catholic population; findings may or may not apply in other contexts or with participants with different cultural backgrounds. Other centers serving patients with different cultures or education levels can adapt our tool to their needs using our freely accessible wiki content. Second, although we reached data saturation, the final DA does not yet address all the issues identified. Third, although we attempted to simplify our DA and improve its graphic design, more work still needs to be done with a specialized linguist and information designer to improve user understanding of our DA and then test its effectiveness in improving the match between what matters most to the informed person and the option chosen, as required by IPDAS. In its current version, our DA prompted few changes to participants' goals of care, but it seems to have increased communication of their preferences to their clinicians which leads us to believe that with some modifications, our DA could support clinicians in the difficult task of engaging critically ill patients in shared decision making about goals of care that are congruent with their values and preferences.

## Conclusion

We developed a context- and culturally-adapted DA for ICU patients facing difficult decisions concerning CPR and invasive mechanical ventilation. This study adds to emerging literature on the use of user-centered design to develop DAs and on the use of wikis to support local adaptation of knowledge tools. Future studies need to address the particular communication and decision-making needs of ICU patients facing goals-of-care decisions. Finally, further work needs to document the impact of using our DA to improve patients’ understanding of CPR and invasive mechanical ventilation and its impact on shared decision making.

## Supporting information

S1 Fig User-centered design development process. (TIF)

S1 Text Research protocol. (PDF)

S2 Text Observation grid used during ethnography and rapid prototyping (English translation). (DOCX)

S3 Text Observation grid used during ethnography and rapid prototyping (original in French). (DOCX)

S4 Text Semi-structured interview questions for intensivists. (DOCX)

S5 Text Final version of the paper DA. (DOCX)

S6 Text Images of the online GO-FAR prediction rule. (DOCX)

S1 Table Performance of the final GO-FAR score. (DOCX)

S2 Table Results of multivariate analysis for final GO-FAR model. (DOCX)

S3 Table Assessment (based on IPDAS) of the original and final decision aids. (DOCX)

## Abbreviations

CPC: Cerebral Performance Category
CPR: Cardio Pulmonary Resuscitation
DA: Decision Aid
GO-FAR: Good Outcome Following Attempted Resuscitation
ICU: Intensive Care Unit
SDM: Shared Decision Making
