# Supplementary figures and images for: Development of a decision aid for cardiopulmonary resuscitation and invasive mechanical ventilation in the intensive care unit employing user-centered design and a wiki platform for rapid prototyping
Source: PLoS One. 2018 Feb 15;13(2):e0191844. doi: 10.1371/journal.pone.0191844 (PMC5813934; doi:10.1371/journal.pone.0191844)

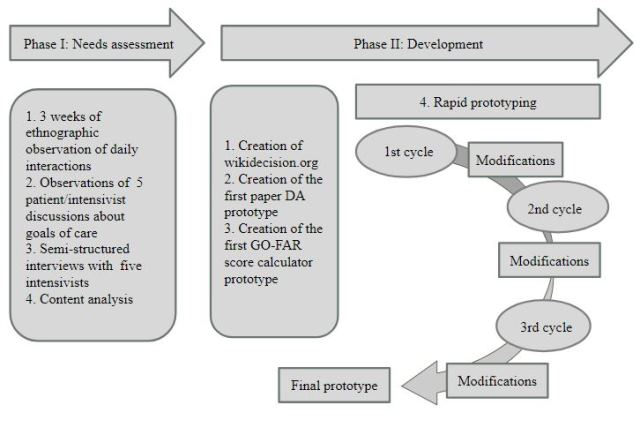

Supplement: S1 Fig — (TIF) [file pone.0191844.s001.tif]

**S6 Text Images of the online GO-FAR prediction rule**


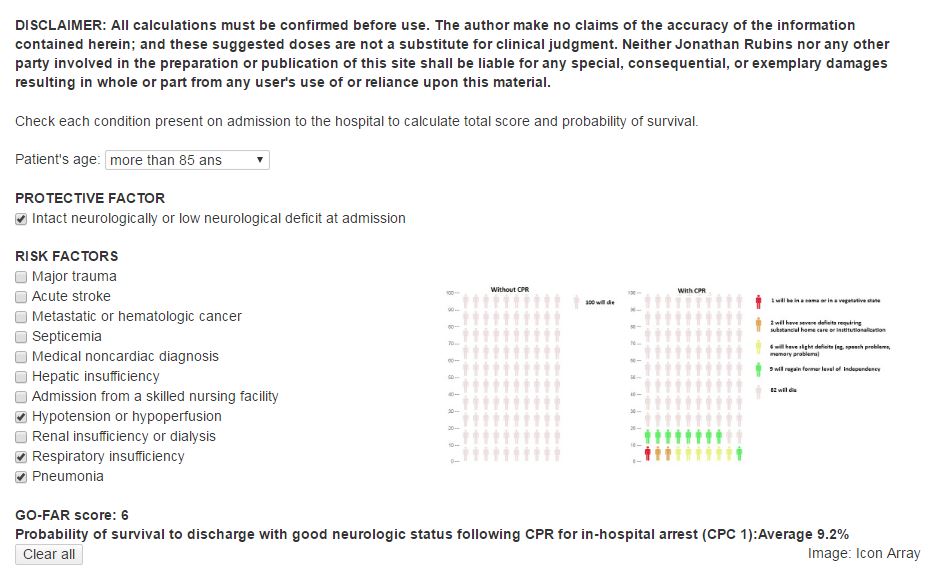


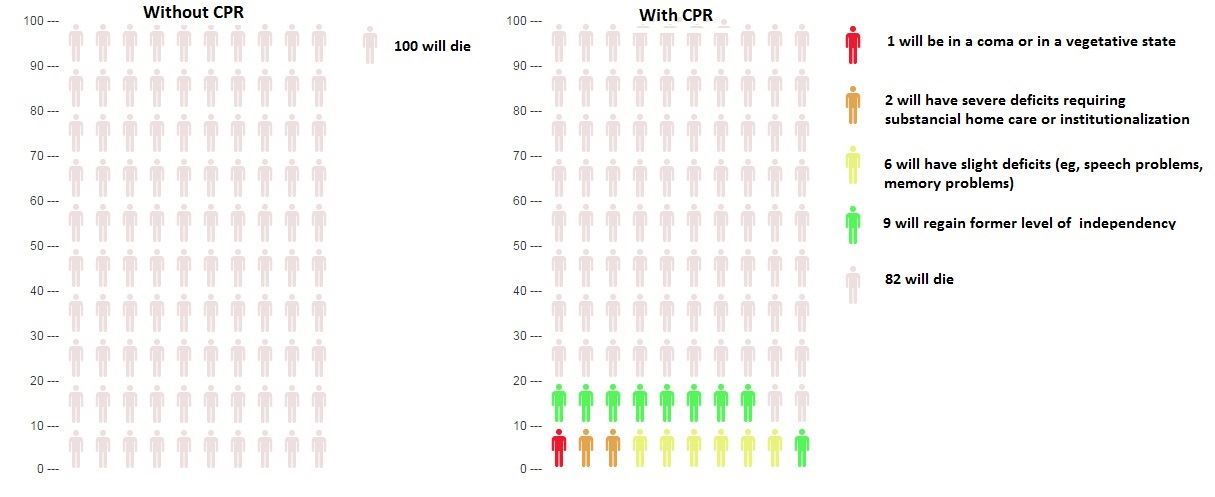

Supplement: S6 Text — (DOCX) [file pone.0191844.s007.docx]
