# Supplementary material for: Development of a decision aid for cardiopulmonary resuscitation and invasive mechanical ventilation in the intensive care unit employing user-centered design and a wiki platform for rapid prototyping
Source: PLoS One. 2018 Feb 15;13(2):e0191844. doi: 10.1371/journal.pone.0191844 (PMC5813934; doi:10.1371/journal.pone.0191844)
Supplement: S1 Text — (PDF) [file pone.0191844.s002.pdf]

Protocol

Development of a Decision Aid for Cardiopulmonary Resuscitation Involving Intensive Care Unit Patients' and Health Professionals' Participation Using User-Centered Design and a Wiki Platform for Rapid Prototyping: A Research Protocol

Ariane Plaisance[1,2], BSc; Holly O Witteman[1,3,4], PhD; Daren Keith Heyland[5,6], PhD; Mark H Ebell[7], PhD (Epidemiology); Audrey Dupuis[2,8], MSc; Carole-Anne Lavoie-Bérard[2], MD; France Légaré[1,4], MD, PhD; Patrick Michel Archambault[2,4,9], MD MSc, FRCPC

[1]Département de médecine sociale et préventive, Faculté de médecine, Université Laval, Québec, QC, Canada
[2]Centre de recherche du Centre hospitalier affilié universitaire Hôtel-Dieu de Lévis, Lévis, QC, Canada
[3]Vice-décanat à la pédagogie et au développement professionnel continu, Faculté de médecine, Université Laval, Québec, QC, Canada
[4]Axe Santé des populations et pratiques optimales en santé, Centre de recherche du CHU de Québec, Québec, QC, Canada
[5]Clinical Evaluation Research Unit, Kingston General Hospital, Kingston, ON, Canada
[6]Department of Medicine, Queen's University, Kingston, ON, Canada
[7]Health Sciences Campus, University of Georgia, Athens, GA, United States
[8]Département d'information et de communication, Faculté des lettres et des sciences humaines, Université Laval, Québec, Québec, QC, Canada
[9]Département de médecine familiale et médecine d'urgence, Faculté de médecine, Université Laval, Québec, QC, Canada

Corresponding Author:
Ariane Plaisance, BSc
Centre de recherche du Centre hospitalier affilié universitaire Hôtel-Dieu de Lévis
143 rue Wolfe
Lévis, QC, G6V 2Y1
Canada
Phone: 1 4189033058
Fax: 1 4189033058
Email: arianeplaisance@gmail.com

Abstract

Background: Cardiopulmonary resuscitation (CPR) is an intervention used in cases of cardiac arrest to revive patients whose heart has stopped. Because cardiac arrest can have potentially devastating outcomes such as severe neurological deficits even if CPR is performed, patients must be involved in determining in advance if they want CPR in the case of an unexpected arrest. Shared decision making (SDM) facilitates discussions about goals of care regarding CPR in intensive care units (ICUs). Patient decision aids (DAs) are proven to support the implementation of SDM. Many patient DAs about CPR exist, but they are not universally implemented in ICUs in part due to lack of context and cultural adaptation. Adaptation to local context is an important phase of implementing any type of knowledge tool such as patient DAs. User-centered design supported by a wiki platform to perform rapid prototyping has previously been successful in creating knowledge tools adapted to the needs of patients and health professionals (eg, asthma action plans). This project aims to explore how user-centered design and a wiki platform can support the adaptation of an existing DA for CPR to the local context.

Objective: The primary objective is to use an existing DA about CPR to create a wiki-based DA that is adapted to the context of a single ICU and tailorable to individual patient's risk factors while employing user-centered design. The secondary objective is to document the use of a wiki platform for the adaptation of patient DAs.

Methods: This study will be conducted in a mixed surgical and medical ICU at Hôtel-Dieu de Lévis, Quebec, Canada. We plan to involve all 5 intensivists and recruit at least 20 alert and oriented patients admitted to the ICU and their family members if available. In the first phase of this study, we will observe 3 weeks of daily interactions between patients, families, intensivists, and other allied health professionals. We will specifically observe 5 dyads of attending intensivists and alert and oriented patients discussing goals of care concerning CPR to understand how a patient DA could support this decision. We will also conduct individual interviews with the 5 intensivists to identify their needs concerning the implementation of a DA. In the second phase

XSL•FO
RenderX

of the study, we will build a first prototype based on the needs identified in Phase I. We will start by translating an existing DA entitled “Cardiopulmonary resuscitation: a decision aid for patients and their families.” We will then adapt this tool to the needs we identified in Phase I and archive this first prototype in a wiki. Building on the wiki’s programming architecture, we intend to integrate the Good Outcome Following Attempted Resuscitation risk calculator into our DA to determine personal risks and benefits of CPR for each patient. We will then present the first prototype to 5 new patient-intensivist dyads. Feedback about content and visual presentation will be collected from the intensivists through short interviews while longer interviews will be conducted with patients and their family members to inform the visual design and content of the next prototype. After each rapid prototyping cycle, 2 researchers will perform qualitative content analysis of data collected through interviews and direct observations. We will attempt to solve all content and visual design issues identified before moving to the next round of prototyping. In all, we will conduct 3 prototyping cycles with a total of 15 patient-intensivist dyads.

Results: We expect to develop a multimedia wiki-based DA to support goals of care discussions about CPR adapted to the local needs of patients, their family members, and intensivists and tailorable to individual patient risk factors. The final version of the DA as well as the development process will be housed in an open-access wiki and free to be adapted and used in other contexts.

Conclusions: This study will shed new light on the development of DAs adapted to local context and tailorable to individual patient risk factors employing user-centered design and a wiki to support rapid prototyping of content and visual design issues.

(JMIR Res Protoc 2016;5(1):e24) doi:10.2196/resprot.5107

KEYWORDS

cardiopulmonary resuscitation; end-of-life planning; goals of care discussions; intensive care medicine; medical informatics; shared decision making; user-centered design; wikis

Introduction

Background

Over the last 90 years, Canadians have gained an average of 24.6 years of life expectancy according to the Conference Board of Canada [1]. Over the same period, the most common place of death has shifted from home to hospital [2]. Therefore, a growing number of older and sicker patients are being admitted to an intensive care unit (ICU) where either they or their family members will have to make crucial decisions about their goals of care [3]. One of these decisions is to decide whether or not to conduct cardiopulmonary resuscitation (CPR) in the event of a cardiac arrest.

Data from the Get with the Guidelines-Resuscitation registry, the world’s largest database on in-hospital cardiopulmonary arrest from over 400 participating hospitals in the United States, estimate that the incidence of treated cardiac arrest is 0.92/1000 patient days. Almost half of these events (48%) occur in the ICU [4]. Over the years, CPR has become a standard intervention used in almost all cases of cardiac or respiratory arrest unless a do-not-resuscitate order (DNR) is recorded in the patient’s chart [5]. However, estimated survival to discharge after treatment for in-hospital cardiac arrest is 18%, with about half of these patients being neurologically intact or having only mild neurologic deficits at discharge [4].

Because cardiac arrest can have potentially devastating outcomes even if CPR is performed, making the decision about whether or not to initiate CPR should involve the patient or his/her surrogate decision maker(s) in cases where the patient is unable to participate in decision making [4]. Discussions about advance directives for CPR in the ICU are a major source of stress and grief for patients, their family members, and their health care professionals. Shared decision making (SDM), a process in which health care professionals and patients work together to make health care choices, offers opportunities to facilitate discussions about advance directives regarding CPR in ICU settings [6].

A common way to support SDM is the use of patient decision aids (DAs). DAs are generally used in situations where a range of treatments are possible, with each treatment having its own advantages and disadvantages. They are intended to be used by patients to complement health care professionals’ counselling. DAs aim to clearly present the best available evidence and to support patients’ reflections about their values and preferences [7].

There exists a series of multiplatform DAs about CPR developed and owned by ACP Decision, an American nonprofit foundation, and adapted to various care settings, illness, and decision makers (patients or surrogate decision makers). Although these DAs are available in multiple languages, there currently lacks a French version that could be used by the population in the Province of Québec where the predominant language is French. Moreover, ACP Decision’s DAs are currently only available to organizations within the United States. We were granted permission to adapt another DA about CPR that was developed in Canada, the “Cardiopulmonary resuscitation: decision aid for patients and their families” [5]. However, in addition to being only available in English, it was initially designed for patients hospitalized in the general hospital ward. Patients admitted to the ICU are critically ill and have specific contextual factors (eg, urgency to make a decision, difficulty reading, unstable condition) that need to be considered in the creation of a DA designed for their use. Moreover, this DA only presents population-level statistics about the outcomes of CPR classified by age category and by the reason of cardiac arrest. It does not present a precise and tailored survival estimate for each patient. The Good Outcome Following Attempted Resuscitation (GO FAR) clinical prediction rule [4] has recently been developed to help health care providers produce better estimates of patients’ likelihood of survival after an episode of in-hospital cardiac arrest, with these patients being neurologically intact or having

XSL•FO
RenderX

only minimal deficits at discharge (Cerebral Performance Category 1 [CPC 1]). The GO FAR clinical prediction rule assigns scores to 11 risk factors such as major trauma, renal insufficiency, and septicemia among others and 1 protection factor (ie, being neurologically intact before cardiac arrest). Using these variables, a final estimate of the survival rate with minimal neurological deficits after in-hospital CPR is generated. The final survival-to-discharge rates with CPC 1 range from a maximum of 27.7% to a minimum of 0.9% [4].

Adapting knowledge tools such as DAs to local context is a crucial step in knowledge translation because it (1) reduces duplication of effort and optimizes use of existing resources; (2) encourages consideration of implementation and "fit" with the local context; (3) enhances applicability; and (4) engages local knowledge users, thus increasing the likelihood of uptake [8]. This step therefore requires engaging local knowledge users to ensure that the knowledge is relevant and applicable to their needs, from its generation to its implementation [9]. Although this is a key step in knowledge translation, little is known about how to accomplish this step effectively [10-12]. Experts have referred to this problem as an "evidence-based crisis" [10,13] and have challenged researchers to find innovative solutions that support the involvement of local knowledge users in adapting knowledge tools to their contexts [14-17].

We submit that using a wiki, a website that can be consulted and edited by anyone who is granted access, could be an effective strategy for adapting DAs to local contexts, as wikis were precisely designed to involve users interactively in the generation and application of knowledge [18]. Wikis—highly accessible, interactive communication vehicles—have also been shown to increase professionals' self-efficacy with regard to their use of various types of knowledge tools [18-20]. The use of a wiki to perform rapid prototyping has successfully helped in the creation of knowledge tools adapted to the needs of patients and health care professionals [18,21-26]; however, to the best of our knowledge, our team is the first to use a wiki to support the creation of a patient DA.

Objectives

The primary objective is to use an existing DA about CPR to create a wiki-based DA that is adapted to the context of a single ICU and tailorable to individual patient risk factors while employing a user-centered design. The secondary objective is to document the use of a wiki platform for the adaptation of knowledge tools such as patient DAs.

Methods

We will employ a user-centered design and a rapid prototyping method to involve patients and other end users in the development process. User-centered design is a longstanding and proven framework and methodology for the development of products, services, and systems [8,27-29]. User-centered design is a highly iterative method for optimizing the user experience—and thus the effectiveness—of a system, service, or product [27]. In this framework, a user is any person who interacts with (in other words, "uses") the system, service, or product for some purpose.

Phase I: Ethnographic Needs Assessment

The main researcher (AP) will conduct 3 weeks of ethnographic observation of patients, families, intensivists, and other allied health professionals' daily interactions. Ethnography is a qualitative method involving the immersion of the researcher into the setting to be studied. Traditionally used in cultural anthropology, ethnography is increasingly being used in health care research. Ethnography allows discovering rich information that could not have been discovered through surveys or other quantitative methods [30]. Because the presence of the main researcher in the ICU will not interfere with the usual care process and that the main goal of these observations is to learn how to improve the decision-making process in the ICU, only verbal consent will be required by the Ethics Review Committee to complete these observations.

During the observation sessions, the main researcher will specifically observe 5 dyads of attending intensivists and alert and oriented ICU-admitted patients, and their family members if available, who need to clarify whether or not to initiate CPR in case of a cardiac arrest. Patients who are unstable, aged less than 18 years, have cognitive impairments, and who do not speak French will be excluded. Potential participants will be identified by attending intensivists. Aptitude of the patients to participate will be confirmed at the beginning of the observation by their attending intensivist using a short evaluation of their orientation in time, space, and person. Using an observation grid developed by a human factors engineer (HW), the main researcher will observe the discussion and note users' needs, goals, strengths, and limitations of the actual process of decision making without the use of a DA. The first 5 eligible patients will be enrolled in the study. The following data will be collected from the patient's chart: date of birth, sex, reason of ICU admission, and level of medical intervention requested on admission to the ICU (full code, limited intervention, DNR, patient undecided or information not available).

In addition to ethnography, the main researcher will conduct one 60-minute semistructured interview with each of the 5 intensivists involved in the project. The development of the interview grid will be informed by the data collected during the ethnography. We will also collect the following demographic information about the 5 intensivists: age, sex, clinical specialty, and number of years working in the ICU.

Phase II: Rapid Prototyping

Development of the First Prototype

The needs gathered in the first phase will inform the construction of the first CPR DA prototype that will be built according to the International Patient Decision Aid Standards [31]. To develop this first prototype, we will start by translating and adapting the existing "Cardiopulmonary resuscitation: a decision aid for patients and their families" [5] using a recognized method for cultural adaptation and translation [32]. This method will require that 2 translators produce 2 translations from English to French. These 2 versions will then be merged and backtranslated to English and presented to the original authors to identify any discrepancies that need to be addressed. We will also translate the GO FAR score [4] into French using the same

XSL•FO
RenderX

method. Using the programming architecture of a wiki platform, we aim to integrate the GO FAR rule into our DA so that patients can personalize the calculation of their own survival rate after CPR. The 5 intensivists and other allied health professionals working in the ICU at Hôtel-Dieu de Lévis, Québec, Canada will be granted access to the wiki platform and will be requested to report any content and visual presentation issues using the collaborative writing functionality of the wiki. Any major issues will be addressed prior to the beginning of the rapid prototyping with actual patients and family members.

Rapid Prototyping Cycles

Rapid prototyping will involve 15 new patients admitted to the ICU and their family members if available. Alert and oriented patients needing to discuss their goals of care or needing to validate goals of care previously discussed will be identified by the main researcher in collaboration with the attending intensivist. A sample of 5 eligible patients will be enrolled at each of the 3 rapid prototyping cycles. Previous uses of this method suggest that a sample of at least 15 participants is adequate to detect over 90% of all usability problems related to products [33].

Inclusion and Exclusion Criteria for Phase II

Patients will be alert, oriented, and capable to consent to participate in our project (inclusion criteria). This will be determined by the attending intensivist. The exclusion criteria are as follows: patients who participated in the first phase, with cognitive impairments and/or critically unstable conditions, aged less than 18 years, and those who do not speak French. Family members of the participating patients will also be invited to participate if available at the time of the study, but this will be optional.

Consent to Participate and Recruitment

Potential participants will be identified by the main researcher who will first obtain consent from the attending intensivist to approach the patient. Although we will purposively attempt to target a wide range of different types of patients based on their age, sex, and disease process, these participants will form a convenience sample based on their availability at the time of recruitment. Once the intensivist judges that the eligible patient is capable and fit to participate, the main researcher will obtain written consent from the patient to participate and will then present the DA to the patient and/or a family member. A minimum of 3 hours will be given to the participant to read the document. A follow-up meeting will be arranged 3 hours later with the attending intensivist so that the patient can provide his/her feedback on our DA and engage in a discussion about their goals of care.

Intervention and Data Collection

The patient/family member and the intensivist will be requested to review together the clinical content and visual aspect of the wiki-based DA. Using an observation grid developed by a human factors engineer (HW), the main researcher will observe users' needs regarding our DA and will assess its strengths and limitations. Then, the main researcher will conduct interviews with the patients and/or their family members. The interview will start with 3 sociodemographic questions about the participants' highest level of education completed, their profession, and their religion. Then, 7 open-ended questions will follow about the DA concerning the following:

- Clarity of information
- Social acceptability of the information presented
- Relevance of the information presented
- Preferred element of the DA
- Suggested improvements to be made to the DA

Finally, each participant will be asked if they had previously discussed their goals of care and their preferences about CPR. We will also verify if our DA changed their previous advance directives (eg, changing from wanting to receive CPR to now refusing CPR). After each use of our DA with any patient, intensivists will also be requested to provide feedback about their experience using the DA with their patient. All interviews will be recorded and then transcribed verbatim.

We will also collect the following data from the patient's chart: date of birth, sex, reason for ICU admission, and the code status preferences upon admission to the ICU.

Data Analysis

After each rapid prototyping cycle, 2 researchers (AP and PA) will perform qualitative content analysis of the verbatim transcripts, audio recordings, and researcher interview notes and observations to identify the usability problems (eg, visual design, format, layout of information), and need for content clarification. Any problem identified will be addressed prior to the next round of user testing. All changes to the prototype will be done online using the wiki platform to keep track of the changes and the different prototype versions produced.

We will perform descriptive statistical analyses of the patient and intensivist sociodemographic data. We will also measure the proportion of patients who state that they changed their goals of care after reading our DA and will note if this change increased the level of care or decreased it. We will also compare patient's final decision about CPR after reading our DA with the level of care documented in the medical chart.

Ethical Considerations

This study was approved by the Research Ethics Board of the Centre Intégré de Santé et de Services Sociaux de Chaudière-Appalaches on January 28, 2015 (CER-1415-019). Informed written consent will be obtained from patients and family members participating in Phase II. Participants will have the opportunity to withdraw from the study at any time. Quantitative data gathered about the participants and qualitative information gathered during the observation sessions and interviews will be kept strictly confidential and the study results will not allow to identify participants. Five years after the publication of the study results, all information about participants will be destroyed. If the participant or a family member of the participant demonstrates by his or her words, gestures, or behavior any kind of discomfort with respect to information contained in the decision tool, the situation will be reported to the attending intensivist. Patients will not be paid for their participation. Because family members participating in the prototyping phase could be asked to travel to the ICU to

XSL•FO
RenderX

meet our research team, they will be offered a small stipend to pay for parking or a meal at the hospital's cafeteria.

Results

We expect to create a wiki-based DA about CPR adapted to the local needs of patients, their family members, and their attending intensivists in a single ICU and tailorable to each patient's characteristics. The final content and visual aspect of the DA will be influenced by the comments received and interactions observed with the 15 dyads in the rapid prototyping phase. This study will be conducted between July and December 2015. We expect that the final version of our DA as well as a detailed description of the steps used to develop it will be available online at the beginning of 2016.

Discussion

Our study proposes a quick, efficient, and low-cost development methodology for DAs adapted to patients' needs and to local care settings. As the population is aging and health care costs are increasing, health systems need a large number of DAs based on constantly evolving evidence. This is even more pertinent in the Province of Quebec with the implementation of and end-of-life law that regulates end-of-life care and advance directives planning [34]. Aside from the more controversial aspects of this law (ie, physician-assisted dying) this law mandates the creation of a province-wide register of patient's advance directives. This register will contain advance directives prepared by patients given by a notarial act or in the presence of a witness on a form approved by the Minister of Health. The creation of this register will increase the need for highly usable and accessible DAs that can support patients' decision making about end-of-life questions such as the one about CPR. We expect that using a wiki will help health professionals and researchers across Quebec, Canada, and elsewhere adapt our DA to their own context and culture, thus reducing duplication and accelerating the dissemination of such DAs for the benefit of more patients.

Thus, this study will enrich the emerging literature on the use of wikis and other collaborative writing applications to create and disseminate knowledge application tools, as well as applying user-centered design to develop DAs that better support SDM.

Limitations

Our study protocol has some limitations. First, although studies on usability testing suggest that 15 patients are sufficient to address most usability issues, we cannot ensure that our final DA will address all the issues about end-of-life decisions that are very complex, emotionally charged, and culturally sensitive. Our wiki platform will however offer an interesting solution to facilitate making improvements to our local DA if new usability or content issues arise in the future. Second, the vast majority of patients admitted to our ICU setting will be white, Catholic, and French-speaking while the issues of end-of-life decisions are deeply linked to culture and religion [35]. This will limit the broader use of our context-adapted DA to settings that are more multicultural. Nevertheless, the use of our wiki platform and methodology will still help other researchers and health professionals to adapt our open-source and free tool to these other cultural settings. Third, although we aim to recruit as many family members as possible to make our DA fully adapted to their needs as well, their participation will be optional for feasibility and ethical reasons. Future studies will have to explore the development of DAs adapted to the needs of surrogate decision makers in situations where patients are unable to make decisions for themselves. Finally, this study will not measure the impact of creating a context-adapted DA. A prospective study assessing the impact of our DA on patient satisfaction, quality of care, and patients' decisional conflict will be needed.

Conclusion

This study will enrich the emerging literature on the use of wikis and other collaborative writing applications to adapt knowledge tools to the local context, as well as applying user-centered design to develop DAs that better support SDM. We expect that using a wiki will help other centers and researchers adapt our DA to their own context and culture, thus reducing duplication and accelerating the dissemination of such DAs.

Acknowledgments

The authors would like to thank the Vice-Décanat à la Recherche et Aux Études Supérieures and the Faculty of Medicine at Université Laval, the Respiratory Health Network of the Fonds de Recherche du Québec-Santé, the Centre Intégré de Santé et de Services Sociaux de Chaudière-Appalaches (Secteur Alphonse-Desjardins) and the Fondation de l'Hôtel-Dieu de Lévis. The authors would also like to thank the nurses, respiratory therapists, and other health care professionals who work in the ICU at l'Hôtel-Dieu de Lévis, Nathalie Boucher, Isabelle Michel, Brian J. Zikmund-Fisher, Michael E. Wilson, Christopher Cox, Carrie-Anna McGinn, Rim Charaf, Dario Gomez, Fatoumata Tounkara, Carmen Irambona-Nyandwi, Samuel Bilodeau, Alexis Turgeon-Fournier, Jean-Michel Constantin, Matthieu Ratthé, Todd Gorman, Jean-François Bellemare, Christine Drouin, Benoît Duhaime, Simon Bordeleau, Ann Laberge, and Amélie Plaisance.

Conflicts of Interest

None declared.

References

XSL•FO
RenderX

1. The Conference Board of Canada. How Canada Performs, Life Expectancy 2015. 2012 Feb. URL: http://www.conferenceboard.ca/hcp/details/health/life-expectancy.aspx [accessed 2016-01-20] [WebCite Cache ID 6egGb7K7V]
2. Fowler R, Hammer M. End-of-life care in Canada. Clin Invest Med 2013;36(3):E127-E132. [Medline: 23739666]
3. Roch A, Wiramus S, Pauly V, Forel J, Guervilly C, Gainnier M, et al. Long-term outcome in medical patients aged 80 or over following admission to an intensive care unit. Crit Care 2011;15(1):R36 [FREE Full text] [doi: 10.1186/cc9984] [Medline: 21261976]
4. Ebell MH, Jang W, Shen Y, Geocadin RG, Get With the Guidelines–Resuscitation Investigators. Development and validation of the Good Outcome Following Attempted Resuscitation (GO-FAR) score to predict neurologically intact survival after in-hospital cardiopulmonary resuscitation. JAMA Intern Med 2013 Nov 11;173(20):1872-1878. [doi: 10.1001/jamainternmed.2013.10037] [Medline: 24018585]
5. Frank C, Pichora D, Suurdt J, Heyland D. Development and use of a decision aid for communication with hospitalized patients about cardiopulmonary resuscitation preference. Patient Educ Couns 2010 Apr;79(1):130-133. [doi: 10.1016/j.pec.2009.08.002] [Medline: 19766436]
6. Kryworuchko J, Hill E, Murray MA, Stacey D, Fergusson DA. Interventions for shared decision-making about life support in the intensive care unit: a systematic review. Worldviews Evid Based Nurs 2013 Feb;10(1):3-16. [doi: 10.1111/j.1741-6787.2012.00247.x] [Medline: 22490044]
7. Légaré F, Stacey D, Turcotte S, Cossi M, Kryworuchko J, Graham ID, et al. Interventions for improving the adoption of shared decision making by healthcare professionals. Cochrane Database Syst Rev 2014;9:CD006732. [doi: 10.1002/14651858.CD006732.pub3] [Medline: 25222632]
8. Searl MM, Borgi L, Chemali Z. It is time to talk about people: a human-centered healthcare system. Health Res Policy Syst 2010;8:35 [FREE Full text] [doi: 10.1186/1478-4505-8-35] [Medline: 21110859]
9. Straus S, Tetroe J, Graham I. Knowledge translation in health care moving from evidence to practice. Chichester, West Sussex: Wiley BMJI Books; 2013.
10. Greenhalgh T, Howick J, Maskrey N, Evidence Based Medicine Renaissance Group. Evidence based medicine: A movement in crisis? BMJ 2014;348:g3725 [FREE Full text] [Medline: 24927763]
11. Gagliardi AR, Alhabib S, members of Guidelines International Network Implementation Working Group. Trends in guideline implementation: a scoping systematic review. Implement Sci 2015;10:54 [FREE Full text] [doi: 10.1186/s13012-015-0247-8] [Medline: 25895908]
12. Gagliardi AR, Brouwers MC, Bhattacharyya OK. The development of guideline implementation tools: a qualitative study. CMAJ Open 2015;3(1):E127-E133 [FREE Full text] [doi: 10.9778/cmajo.20140064] [Medline: 25844365]
13. Pronovost PJ. Enhancing physicians' use of clinical guidelines. JAMA 2013 Dec 18;310(23):2501-2502. [doi: 10.1001/jama.2013.281334] [Medline: 24310916]
14. McKillop A, Crisp J, Walsh K. Practice guidelines need to address the 'how' and the 'what' of implementation. Prim Health Care Res Dev 2012 Jan;13(1):48-59. [doi: 10.1017/S1463423611000405] [Medline: 22008308]
15. Dobbins M, Hanna SE, Ciliska D, Manske S, Cameron R, Mercer SL, et al. A randomized controlled trial evaluating the impact of knowledge translation and exchange strategies. Implement Sci 2009;4:61 [FREE Full text] [doi: 10.1186/1748-5908-4-61] [Medline: 19775439]
16. Shekelle PG, Kravitz RL, Beart J, Marger M, Wang M, Lee M. Are nonspecific practice guidelines potentially harmful? A randomized comparison of the effect of nonspecific versus specific guidelines on physician decision making. Health Serv Res 2000 Mar;34(7):1429-1448 [FREE Full text] [Medline: 10737446]
17. Grilli R, Lomas J. Evaluating the message: the relationship between compliance rate and the subject of a practice guideline. Med Care 1994 Mar;32(3):202-213. [Medline: 8145598]
18. Archambault PM, van de Belt Tom H, Grajales FJ, Faber MJ, Kuziemsky CE, Gagnon S, Nelen Willianne L D M, Kremer Jan A M, et al. Wikis and collaborative writing applications in health care: a scoping review. J Med Internet Res 2013;15(10):e210 [FREE Full text] [doi: 10.2196/jmir.2787] [Medline: 24103318]
19. McLean R, Richards BH, Wardman JI. The effect of Web 2.0 on the future of medical practice and education: Darwikinian evolution or folksonomic revolution? Med J Aust 2007 Aug 6;187(3):174-177. [Medline: 17680746]
20. Eysenbach G. Medicine 2.0: social networking, collaboration, participation, apomediation, and openness. J Med Internet Res 2008;10(3):e22 [FREE Full text] [doi: 10.2196/jmir.1030] [Medline: 18725354]
21. Archambault PM. WikiBuild: a new application to support patient and health care professional involvement in the development of patient support tools. J Med Internet Res 2011;13(4):e114 [FREE Full text] [doi: 10.2196/jmir.1961] [Medline: 22155746]
22. Archambault PM, Gagnon S, Gagnon M, Turcotte S, Lapointe J, Fleet R, et al. Development and validation of questionnaires exploring health care professionals' intention to use wiki-based reminders to promote best practices in trauma. JMIR Res Protoc 2014;3(3):e50 [FREE Full text] [doi: 10.2196/resprot.3762] [Medline: 25281856]
23. Archambault PM, van de Belt Tom H, Grajales Iii Francisco J, Eysenbach G, Aubin K, Gold I, Kremer Jan A M, et al. Wikis and collaborative writing applications in health care: a scoping review protocol. JMIR Res Protoc 2012;1(1):e1 [FREE Full text] [doi: 10.2196/resprot.1993] [Medline: 23612481]

XSL•FO
RenderX

24. Archambault PM, Légaré F, Lavoie A, Gagnon M, Lapointe J, St-Jacques S, et al. Healthcare professionals' intentions to use wiki-based reminders to promote best practices in trauma care: A survey protocol. Implement Sci 2010;5:45 [FREE Full text] [doi: 10.1186/1748-5908-5-45] [Medline: 20540775]
25. van de Belt Tom H, Faber MJ, Knijnenburg José M L, van Duijnhoven Noortje T L, Nelen Willianne L D M, Kremer Jan A M. Wikis to facilitate patient participation in developing information leaflets: first experiences. Inform Health Soc Care 2014 Mar;39(2):124-139. [doi: 10.3109/17538157.2013.872107] [Medline: 24517459]
26. Gupta S, Wan FT, Newton D, Bhattacharyya OK, Chignell MH, Straus SE. WikiBuild: A new online collaboration process for multistakeholder tool development and consensus building. J Med Internet Res 2011;13(4):e108 [FREE Full text] [doi: 10.2196/jmir.1833] [Medline: 22155694]
27. Witteman HO, Dansokho SC, Colquhoun H, Coulter A, Dugas M, Fagerlin A, et al. User-centered design and the development of patient decision aids: protocol for a systematic review. Syst Rev 2015;4:11 [FREE Full text] [doi: 10.1186/2046-4053-4-11] [Medline: 25623074]
28. Elkin PL. Human Factors Engineering in HI: So What? Who Cares? and What's in It for You? Healthc Inform Res 2012 Dec;18(4):237-241 [FREE Full text] [doi: 10.4258/hir.2012.18.4.237] [Medline: 23346473]
29. Gurses AP, Ozok AA, Pronovost PJ. Time to accelerate integration of human factors and ergonomics in patient safety. BMJ Qual Saf 2012 Apr;21(4):347-351. [doi: 10.1136/bmjqs-2011-000421] [Medline: 22129929]
30. Goodson L, Vassar M. An overview of ethnography in healthcare and medical education research. J Educ Eval Health Prof 2011;8:4 [FREE Full text] [doi: 10.3352/jeehp.2011.8.4] [Medline: 21637319]
31. Elwyn G, O'Connor A, Stacey D, Volk R, Edwards A, Coulter A, International Patient Decision Aids Standards (IPDAS) Collaboration. Developing a quality criteria framework for patient decision aids: online international Delphi consensus process. BMJ 2006 Aug 26;333(7565):417 [FREE Full text] [doi: 10.1136/bmj.38926.629329.AE] [Medline: 16908462]
32. Beaton DE, Bombardier C, Guillemin F, Ferraz MB. Guidelines for the process of cross-cultural adaptation of self-report measures. Spine (Phila Pa 1976) 2000 Dec 15;25(24):3186-3191. [Medline: 11124735]
33. Faulkner L. Beyond the five-user assumption: Benefits of increased sample sizes in usability testing. Behav Res Methods Instrum Comput 2003 Aug;35(3):379-383. [Medline: 14587545]
34. Gouvernement du Québec. Loi Concernant les Soins de fin de vie. URL: http://www2.publicationsduquebec.gouv.qc.ca/dynamicSearch/telecharge.php?type=2&file=%2F%2FS_32_0001%2FS32_0001.htm [accessed 2016-02-02] [WebCite Cache ID 6egH5BNAR]
35. Lu C, Johantgen M. Factors associated with treatment restriction orders and hospice in older nursing home residents. J Clin Nurs 2011 Feb;20(3-4):377-387. [doi: 10.1111/j.1365-2702.2010.03346.x] [Medline: 21118320]

Abbreviations

CPC 1: Cerebral Performance Category 1
CPR: cardiopulmonary resuscitation
DA: decision aid
DNR: do-not-resuscitate order
GO FAR: Good Outcome Following Attempted Resuscitation
ICU: intensive care unit
SDM: shared decision making

Edited by G Eysenbach; submitted 04.09.15; peer-reviewed by A Jamal, A Syrowatka; comments to author 30.09.15; revised version received 09.12.15; accepted 04.01.16; published 11.02.16

Please cite as:
Plaisance A, Witteman HO, Heyland DK, Ebell MH, Dupuis A, Lavoie-Bérard CA, Légaré F, Archambault PM
Development of a Decision Aid for Cardiopulmonary Resuscitation Involving Intensive Care Unit Patients' and Health Professionals' Participation Using User-Centered Design and a Wiki Platform for Rapid Prototyping: A Research Protocol
JMIR Res Protoc 2016;5(1):e24
URL: http://www.researchprotocols.org/2016/1/e24/
doi:10.2196/resprot.5107
PMID:26869137

©Ariane Plaisance, Holly O. Witteman, Daren Keith Heyland, Mark H. Ebell, Audrey Dupuis, Carole-Anne Lavoie-Bérard, France Légaré, Patrick Michel Archambault. Originally published in JMIR Research Protocols (http://www.researchprotocols.org), 11.02.2016. This is an open-access article distributed under the terms of the Creative Commons Attribution License (http://creativecommons.org/licenses/by/2.0/), which permits unrestricted use, distribution, and reproduction in any medium,

XSL•FO
RenderX

provided the original work, first published in JMIR Research Protocols, is properly cited. The complete bibliographic information, a link to the original publication on http://www.researchprotocols.org, as well as this copyright and license information must be included.

XSL•FO
RenderX
