# Supplementary material for: Development of a decision aid for cardiopulmonary resuscitation and invasive mechanical ventilation in the intensive care unit employing user-centered design and a wiki platform for rapid prototyping
Source: PLoS One. 2018 Feb 15;13(2):e0191844. doi: 10.1371/journal.pone.0191844 (PMC5813934; doi:10.1371/journal.pone.0191844)
Supplement: S2 Text — (DOCX) [file pone.0191844.s003.docx]

**S2 Text Observation grid used during ethnography and rapid prototyping (English translation)**

| Patient record number:  Clinician:  Observers:  Observation start time:  Observation end time: | Exclusion criteria:  Known dementia  Urgent decision to be made  Inclusion criteria:  Patient capable and alert  Patient admitted to the ICU |
| --- | --- |
| Sociodemographic information  Age:  Sex:  Education level:  Religion: | |
| Patients | Families |
| Is the information presented clear? | |
|  |  |
| Does the information presented shock you, upset you? | |
|  |  |
| Do you feel that the information presented in relevant? | |
|  |  |
| What is your favourite aspect of the tool? | |
|  |  |
| What would you improve? | |
|  |  |
| Context (environment, etc.) | |
|  |  |
| Other observations | |
|  |  |
