# Supplementary material for: Development of a decision aid for cardiopulmonary resuscitation and invasive mechanical ventilation in the intensive care unit employing user-centered design and a wiki platform for rapid prototyping
Source: PLoS One. 2018 Feb 15;13(2):e0191844. doi: 10.1371/journal.pone.0191844 (PMC5813934; doi:10.1371/journal.pone.0191844)
Supplement: S3 Text — (DOCX) [file pone.0191844.s004.docx]

**S3 Text Observation grid used during ethnography and rapid prototyping (Original in French)**

Grille d’observation de la consultation

| Numéro de dossier du patient :  Clinicien :  Observateurs :  Heure du début de l’observation:  Heure de la fin de l’observation:  Enregistrement no. : | Critères d’exclusion:  Condition urgente ☐  État d’immunosuppression ☐  Troubles cognitifs ☐  État d’intoxication ☐  Critères d’inclusion:  Patient en mesure lire, écrire et comprendre le français ☐  Patient ayant donné son consentement ☐ |
| --- | --- |

| Contexte |
| --- |
|  |
| Difficultés rencontrées |
|  |
| Autres observations |
|  |

Questionnaire semi-structuré sur l’usage du prototype

| Est-ce que l’information présentée est claire? |
| --- |
|  |
| Est-ce que l’information présentée vous choque, vous heurte? |
|  |
| Est-ce que vous trouvez que l’information présentée est pertinente? |
|  |
| Quel est votre aspect favori de l’outil? |
|  |
| Qu’est-ce que vous voudriez améliorer? |
|  |

Copy of questionnaire used in the study in the original language (French) (S2 Appendix in the Manuscript)

Grille d’entrevue pour les intensivistes

- Quelles sont vos responsabilités en tant que chef de service de l’USI?
- Généralement, est-ce que les patients admis à l’USI ont un niveau de soins? Est-ce que vous croyez que le niveau de soins a été déterminé de manière éclairée?
- Est-ce que vous jugez que les patients et leurs proches sont suffisamment équipés pour prendre une décision éclairée?
- Est-ce que vous jugez que vous être suffisamment équipés pour accompagner les patients dans leurs décisions?
- Y a-t-il des outils disponibles qui peuvent vous aider à informer les patients et leurs proches dans le but qu’ils prennent une décision éclairée?
