# Supplementary material for: Development of a decision aid for cardiopulmonary resuscitation and invasive mechanical ventilation in the intensive care unit employing user-centered design and a wiki platform for rapid prototyping
Source: PLoS One. 2018 Feb 15;13(2):e0191844. doi: 10.1371/journal.pone.0191844 (PMC5813934; doi:10.1371/journal.pone.0191844)
Supplement: S4 Text — (DOCX) [file pone.0191844.s005.docx]

**S4 Text Semi-structured interview questions for intensivists**

- As an intensivist, what are your responsibilities regarding determination of levels of care?

- Generally speaking, do the patients admitted to the ICU have a level of care indicated in their charts? If so, do you believe that (this) level of care choice was determined in an informed manner?

- Do you feel that the patients and their caregivers are sufficiently equipped to make an informed decision about the level lof care they desire?

- Do you feel that you are sufficiently equipped to accompany patients in making their decision?

- Are there any tools available that could help you communicate with patients and their caregivers so that they can make an informed decision?
