# Supplementary material for: Development of a decision aid for cardiopulmonary resuscitation and invasive mechanical ventilation in the intensive care unit employing user-centered design and a wiki platform for rapid prototyping
Source: PLoS One. 2018 Feb 15;13(2):e0191844. doi: 10.1371/journal.pone.0191844 (PMC5813934; doi:10.1371/journal.pone.0191844)
Supplement: S5 Text — (DOCX) [file pone.0191844.s006.docx]

**S5 Text Final version of the paper DA**

**Patient Decision Aid:**

**Sharing Goals for ICU care**


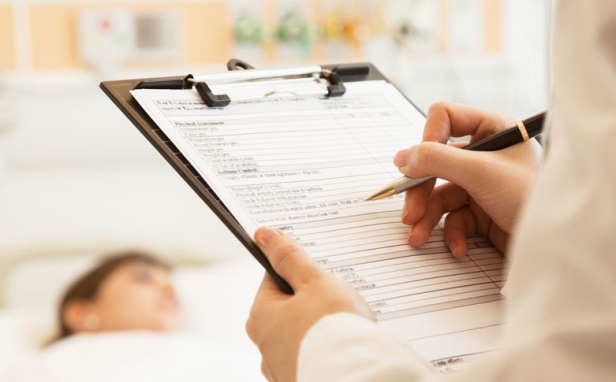


**For patients and families in the Intensive Care Unit (ICU)**

**Introduction**

In the ICU, it is important that we understand your goals of care to ensure that the care we provide meets your health needs and respects your wishes. There are four different goals of care.


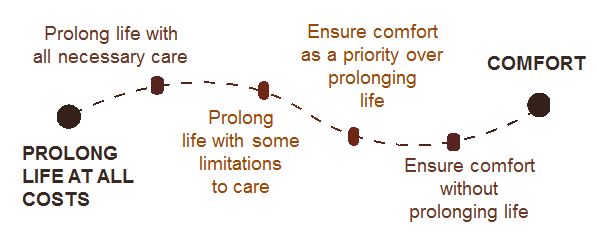


Your physician will meet you to discuss your wishes, current and anticipated levels of independence. Together, you will make a decision about ICU Care, including:

1: Cardiopulmonary Resuscitation (or CPR)

2: Invasive mechanical ventilation (being connected to a breathing machine)

**Your wishes and current and anticipated levels of independence**

**Below are a few questions to help your care team get to know you:**

Do you know anyone who has had CPR or invasive mechanical ventilation ?

What are your wishes when it comes to CPR and invasive mechanical ventilation?

Before your current hospitalization, how did you complete your day-to-day activities*? (Check all that apply)*

|  | 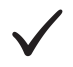 |
| --- | --- |
| without help? |  |
| with an assisting device?  (ex: a cane, a walker) |  |
| with adaptation?  (ex: wheelchair ramps) |  |
| with human help?  (ex: help from a family member) |  |

If you lost your independence after this hospitalization, where would you be willing to live? *(check all that apply)*

|  | 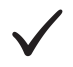 |
| --- | --- |
| at home with help? |  |
| in a long-term care facility for semi-independent persons? |  |
| in a long-term care facility for non-independent persons? |  |

When would prolonging your life become unacceptable? *(you can choose all that apply)*

|  | 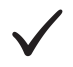 |
| --- | --- |
| If I could not communicate with others |  |
| If I needed help with personal care |  |
| If I were bedridden |  |

Had you made a decision before reading this document:

|  | Yes | No |
| --- | --- | --- |
| about CPR ? |  |  |
| about invasive mechanical ventilation? |  |  |
| about when prolonging your life would be unacceptable? |  |  |

Have you written these wishes somewhere? (ex: a living will)

**Intervention #1 Cardiopulmonary Resuscitation (CPR)**

Cardiopulmonary resuscitation (CPR) is a group of interventions used to try and restart the heart when it stops beating.


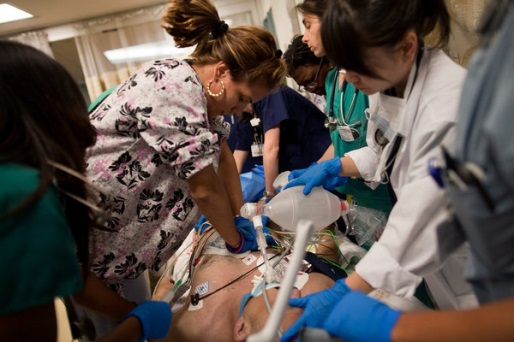


When CPR is performed in a hospital:

1) Blood circulation is maintained by **pressing on the chest**

2) A tube is inserted in the lungs through the mouth to assist breathing (**intubation** and **invasive mechanical ventilation**)

3) Electric shocks (**defibrillation**) can be used

4) Medication is administered

**What are the benefits and the risks of Cardiopulmonary Resuscitation (CPR)?**

If a person’s heart stops beating and nothing is done, the person dies. CPR can provide between 0 to 30% chance of survival, depending on their medical condition¹.

| **BENEFITS** | **RISKS** |
| --- | --- |
| Can avoid a sudden death | Brain injury |
| Can possible regain former level of independence and leave the hospital | Fractured ribs |

Your physician will be able to explain your chance of survival and your anticipated level of independence following CPR.

^1^ Ebell *et al.*, 2014 et Canadian Researchers at the End-of-Life Network

**Intervention #2 Invasive Mechanical Ventilation**

Invasive mechanical ventilation is a machine that pushes air into the lungs through a tube in the month or neck. This is also known as “being kept on life support”.


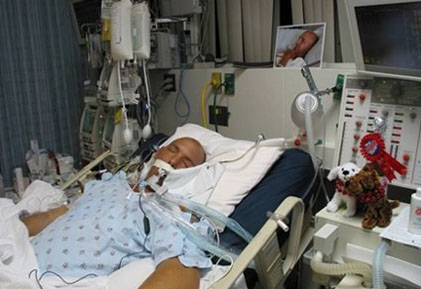


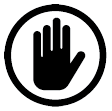


Invasive mechanical ventilation does not heal breathing problem. It keeps the patient alive so that the care team can address the breathing problem. During invasive mechanical ventilation, nurses administer medication to the patient to reduce their discomfort. When a patient is mechanically ventilated, they cannot eat or speak.

**What are the benefits and the risks of Invasive Mechanical Ventilation?**

People kept alive with invasive mechanical ventilation can still die from their illness. While the chances of surviving are higher, some survivors live with important side effects.

| **BENEFITS** | **RISKS** |
| --- | --- |
| Can avoid sudden death | Complications (ex: pneumonia) |
| Can possibly regain former level of independence and leave the hospital | Emotional and physical suffering |

If invasive mechanical ventilation cannot prolong life or is not wanted, other less aggressive and less effective therapies could be offered to you.

**Summary**

This document presented you with information about two ICU interventions. You reflected on your wishes and your current and anticipated levels of independence.


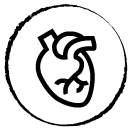
 For now, if your heart stops beating, would you like your care team to attempt CPR?

Yes  No  Undecided


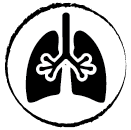
 For now, if you can’t breathe without help, do you want invasive mechanical ventilation?

**Yes  No  Undecided**

Notes

**What happens after this conversation?**

Your goals of care will be added to your chart and your team will work with you to plan your care.


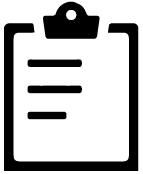


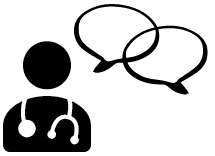


When treatments such as invasive mechanical ventilation or CPR cannot prolong life or are not wanted, you will continue to receive the best care.

You can always change your mind. Just let a team member know.

**This decision aid was developed with patients and family members at Hôtel-Dieu de Lévis ICU**


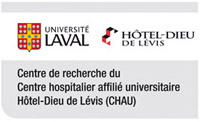

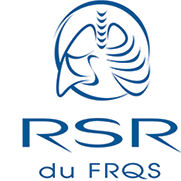


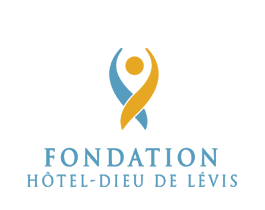


Updated April, 28th, 2017
