# Supplementary material for: Development of a decision aid for cardiopulmonary resuscitation and invasive mechanical ventilation in the intensive care unit employing user-centered design and a wiki platform for rapid prototyping
Source: PLoS One. 2018 Feb 15;13(2):e0191844. doi: 10.1371/journal.pone.0191844 (PMC5813934; doi:10.1371/journal.pone.0191844)
Supplement: S1 Table — (DOCX) [file pone.0191844.s008.docx]

**S1 Table Performance of the Final GO-FAR Score***

| **Performance of the Final GO-FAR Score** | | | |
| --- | --- | --- | --- |
| Risk groups | GO-FAR score category ¹ | Patients in risk category, % | Survivors with CPC score of 1/all with this score (%) |
| Very low (<1%) | ≥ 24 | 9.4 | 37/4799 (0.9) |
| Low (1% to 3%) | 14 to 23 | 19.0 | 194/9725 (2.0) |
| Average (>3% to 15%) | -15 to 13 | 53.6 | 2531/27 464 (9.2) |
| Above average (>15%) | -15 to -6 | 18.1 | 2568/9253 (27.8) |

Abbreviations: CPC, Cerebral Performance Category; GO-FAR, Good Outcome Following Attempted Resuscitation

*Table provided by the author (MME)
