# Supplementary material for: Development of a decision aid for cardiopulmonary resuscitation and invasive mechanical ventilation in the intensive care unit employing user-centered design and a wiki platform for rapid prototyping
Source: PLoS One. 2018 Feb 15;13(2):e0191844. doi: 10.1371/journal.pone.0191844 (PMC5813934; doi:10.1371/journal.pone.0191844)
Supplement: S2 Table — (DOCX) [file pone.0191844.s009.docx]

**S2 Table Results of multivariate analysis for final GO-FAR model***

| **Results of multivariate analysis for final model¹** | |
| --- | --- |
| Variable | GO-FAR score² |
| Neurologically intact or with minimal deficit at admission² | -15 |
| Major trauma | 10 |
| Acute stroke | 8 |
| Metastatic or hematologic cancer | 7 |
| Septicemia | 7 |
| Medical noncardiac diagnosis | 7 |
| Hepatic insufficiency | 6 |
| Admit from skilled nursing facility | 6 |
| Hypotension or hypoperfusion | 5 |
| Renal insufficiency or dialysis | 4 |
| Respiratory insufficiency | 4 |
| Pneumonia | 1 |
| Age, y | |
| 70-74 | 2 |
| 75-79 | 5 |
| 80-84 | 6 |
| ≥ 85 | 11 |

Abbreviations: CPC, Cerebral Performance Category; GO-FAR, Good Outcome Following Attempted Resuscitation

¹ Survival to discharge with a CPC score of 1 is the dependant variable

² Points for GO-FAR scores were assigned based on β coefficients

³ CPC score of 1 at admission

*Table provided by the author (MHE)
