# Supplementary material for: Development of a decision aid for cardiopulmonary resuscitation and invasive mechanical ventilation in the intensive care unit employing user-centered design and a wiki platform for rapid prototyping
Source: PLoS One. 2018 Feb 15;13(2):e0191844. doi: 10.1371/journal.pone.0191844 (PMC5813934; doi:10.1371/journal.pone.0191844)
Supplement: S3 Table — (DOCX) [file pone.0191844.s010.docx]

**S3 Table Assessment (based on IPDAS) of the original and final decision aids**

|  |  | **Original DA** | **Final DA** |
| --- | --- | --- | --- |
| Content | | | |
| 1. | The decision aid describes the condition (health or other) related to the decision. | Yes | Yes |
| 2. | The decision aid describes the decision that needs to be considered (the index decision). | Yes | Yes |
| 3. | The decision aid lists the options (health care or other). | Yes | Yes |
| 4. | The decision aid describes what happens in the natural course of the condition (health or other) if no action is taken. | Yes | Yes |
| 5. | The decision aid has information about the procedures involved (e.g. what is done before, during, and after the health care option). | Yes | Yes |
| 6. | The decision aid has information about the positive features of the options (e.g. benefits, advantages). | Yes | Yes |
| 7. | The decision aid has information about negative features of the options (e.g. harms, side effects, disadvantages). | Yes | Yes |
| 8. | The information about outcomes of options (positive and negative) includes the chances they may happen. | No | Yes/No¹ |
| 9. | The decision aid has information about what the test is designed to measure. | NA | NA |
| 10. | The decision aid describes possible next steps based on the test results. | NA | NA |
| 11. | The decision aid has information about the chances of disease being found with and without screening. | NA | NA |
| 12. | The decision aid has information about detection and treatment of disease that would never have caused problems if screening had not been done. | NA | NA |
| 13. | The decision aid presents probabilities using event rates in a defined group of people for a specified time. | NA | NA |
| 14. | The decision aid compares probabilities (e.g. chance of a disease, benefit, harm, or side effect) of options using the same denominator | NA | NA |
| 15. | The decision aid compares probabilities of options over the same period of time | NA | NA |
| 16. | The decision aid uses the same scales in diagrams comparing options. | NA | NA |
| 17. | The decision aid asks people to think about which positive and negative features of the options matter most to them | Yes | Yes |
| 18. | The decision aid makes it possible to compare the positive and negative features of the available options. | Yes | Yes |
| 19. | The decision aid shows the negative and positive features of the options with equal detail. | Yes | Yes |
| **Development process** | | | |
| 20. | Users (people who previously faced the decision) were asked what they need to prepare them to discuss a specific decision. | Yes | Yes |
| 21. | The decision aid was reviewed by people who previously faced the decision who were not involved in its development and field testing. | Yes | Yes |
| 22. | People who were facing the decision field tested the decision aid. | Yes | Yes |
| 23. | Field testing showed that the decision aid was acceptable to users (the general public & practitioners). | Yes | Yes |
| 24. | Field testing showed that people who were undecided felt that the information was presented in a balanced way. | Yes | Yes |
| 25. | The decision aid provides references to scientific evidence used. | No | Yes |
| 26. | The decision aid reports the date when it was last updated. | No | Yes |
| 27. | The decision aid reports whether authors of the decision aid or their affiliations stand to gain or lose by choices people make after using the decision aid. | Unknown | No |
| 28. | The decision aid (or available technical document) reports readability levels. | No | No |
| **Effectiveness** | | | |
| 29. | There is evidence that the decision aid (or one based on the same template) helps people know about the available options and their features. | Unknown | Unknown |
| 30. | There is evidence that the decision aid (or one based on the same template) improves the match between the features that matter most to the informed person and the option that is chosen. | Unknown | Unknown |
| ¹ Yes: first intervention (CPR); No: second intervention (Invasive mechanical ventilation | | | |

|  |
| --- |
